# Information Analysis on Neural Tuning in Dorsal Premotor Cortex for Reaching and Grasping

**DOI:** 10.1155/2013/730374

**Published:** 2013-05-27

**Authors:** Yan Cao, Yaoyao Hao, Yuxi Liao, Kai Xu, Yiwen Wang, Shaomin Zhang, Qiaosheng Zhang, Weidong Chen, Xiaoxiang Zheng

**Affiliations:** ^1^Qiushi Academy for Advanced Studies, Zhejiang University, Hangzhou 310027, China; ^2^Department of Biomedical Engineering, Zhejiang University, Hangzhou 310027, China; ^3^Key Laboratory of Biomedical Engineering of Ministry of Education, Zhejiang University, Hangzhou 310027, China

## Abstract

Previous studies have shown that the dorsal premotor cortex (PMd) neurons are relevant to reaching as well as grasping. In order to investigate their specific contribution to reaching and grasping, respectively, we design two experimental paradigms to separate these two factors. Two monkeys are instructed to reach in four directions but grasp the same object and grasp four different objects but reach in the same direction. Activities of the neuron ensemble in PMd of the two monkeys are collected while performing the tasks. Mutual information (MI) is carried out to quantitatively evaluate the neurons' tuning property in both tasks. We find that there exist neurons in PMd that are tuned only to reaching, tuned only to grasping, and tuned to both tasks. When applied with a support vector machine (SVM), the movement decoding accuracy by the tuned neuron subset in either task is quite close to the performance by full ensemble. Furthermore, the decoding performance improves significantly by adding the neurons tuned to both tasks into the neurons tuned to one property only. These results quantitatively distinguish the diversity of the neurons tuned to reaching and grasping in the PMd area and verify their corresponding contributions to BMI decoding.

## 1. Introduction

Motor brain-machine interfaces (mBMIs) interpret the motor intents from neural signals to control the external devices, such as the computer curser and the robot arm [1–5]. In the previous studies, the subjects, usually nonhuman primates, are required to manipulate a joy stick in a 2D plane or 3D space to track the target, which mostly focus on the arm movement [6, 7]. Thanks to the occurrence of the artificial prostheses with multi-degrees of freedom and the stable recoding systems, we could step into more complicated paradigms. A few researchers have started to work on elaborate grasping tasks [8–10], which involve the movement of fingers with different gestures.

Previous studies have shown that there are several cortical areas relevant to the reaching and the grasping movements, such as the primary motor cortex (M1), the ventral premotor cortex (PMv), and the dorsal premotor cortex (PMd) [10–13]. Specially, neurophysiological studies have manifested that the PMd area mostly relates to the proximal arm movements [14, 15]. Some neurons fire more frequently when moving towards the preferred direction. Burnod et al. have found that when the initial arm position varied across the working space, the directional preferences of the PMd neurons changed significantly [14]. Messier and Kalaska have proposed a bootstrapping method to estimate the probability that neurons were tuned based on the intertrial variability when the monkey was performing a whole-arm reaching movement in a plane. Significant directional tuning of the neurons in PMd was relatively constant throughout the trials [15]. But recently, some studies have reported that there are also some neurons related to grasping in PMd [16, 17]. Raos et al. firstly found that when the monkey was grasping different shaped objects, the recorded neuron firings in PMd were sensitive for a preferred type of shape [16, 17]. Some researchers suggest that in the PMd area the neural representations of reaching are not completely separated from grasping [17, 18], that is, there are grasp-related neurons in this area. This phenomenon indicates the existence of the neurons tuned to both reaching and grasping tasks. To study their combined representation, Stark et al. performed a mixed paradigm which required the monkey to grasp one of three different objects in six directions [12]. He found that there are neurons related to reaching as well as grasping in PMd. However, the paradigm in [12] combined reaching and grasping in the same trial, which may influence each other because the reaching is always preshaped by the grasping gesture.

In this paper, we are interested in studying the tuning property of the PMd neurons related to reaching and grasping, respectively. Two experimental paradigms are designed to separate these two factors. Two monkeys are instructed to reach in four directions but grasp the same object in the first experiment, named as “Reaching task.” In the second experiment, the monkeys are instructed to grasp four different objects with different gestures but reach in the same direction, named as “Grasping task.” Activities of neuron ensemble in PMd are collected while performing the tasks. We propose to use mutual information (MI) to quantitatively discriminate the neurons tuned to reaching and grasping, respectively. In order to verify the contribution of the tuned neurons in PMd, a support vector machine (SVM) is implemented to decode the activities of the tuned neurons to the corresponding movement and compared with the performance by full ensemble in both reaching and grasping tasks. The decoding is further used to validate if there exist neurons tuned to both tasks, and how much they contribute to the motor decoding. The experimental setup and data acquisition are shown in Section 2.1, followed by the introduction of the mutual information method measuring the neurons' tuning characteristic and the implementation of the decoding algorithm SVM. Results are shown and explained in Section 3. Conclusion and discussion are in Section 4.

## 2. Materials and Methods

### 2.1. Experimental Setup

**Figure 1:**
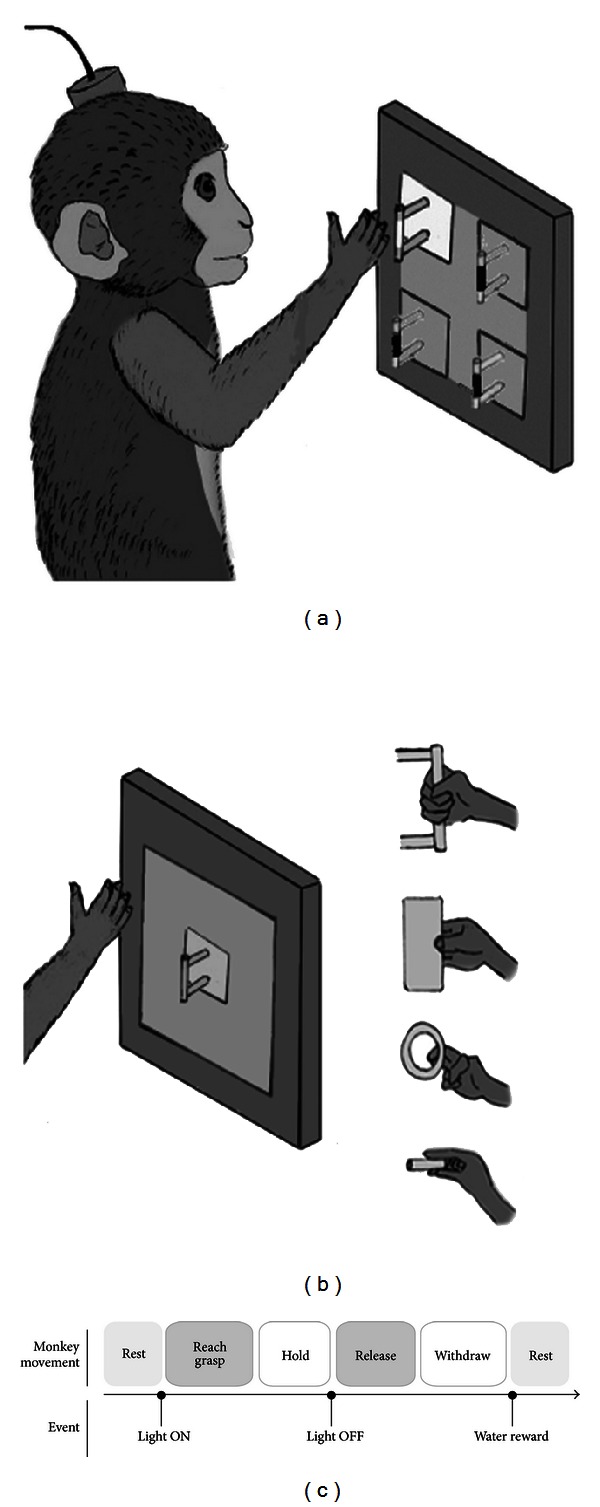
Experimental setup and the trial sequence. (a) The Reaching task. The monkeys were trained to grasp the identical objects in four directions. (b) The Grasping task. The monkeys were trained to grasp four different objects, namely, a small cylinder, a rectangle plate, a ring, and a cone, in the same direction. (c) The time sequence of a single trial.

The paradigm of motor brain-machine interface was designed and implemented in Qiushi Academy for Advanced Studies at Zhejiang University. Two rhesus macaques (male), named B03 and B04, were trained separately to perform two different tasks: the Reaching task and the Grasping task with one of their dominant hands (right hand for B03 and left hand for B04), as illustrated in Figures 1(a) and 1(b). In the Reaching task, four identical objects are fixed on the four corners of a transparent resin glass board, and the monkeys are required to stretch out to grasp the target object in different directions. While in the Grasping task, one object with a certain shape is fixed on the same position of the board (indicating the same direction), and the monkeys are required to grasp it with a specific gesture in each trial. There are four objects in our experiment, namely, a small cylinder, a rectangle plate, a ring, and a cone. One object is grasped for a certain number of trials and changed to another.

During the task, the monkey sits in a primate chair with his head fixed. An LCD monitor is mounted behind the board to illuminate the target object area, as a cue to instruct the monkeys to start the trial. The trial sequence is shown in Figure 1(c). 

The monkey sits in darkness during the intertrial interval (1~2.5 s) with his hand resting on the clapboard. When the cue is on (Light ON), the monkey is required to stretch out and grasp the target object within 600 ms, and hold it for 1~2 s until the cue is off (Light OFF). After Light OFF, the monkey releases the object and withdraws his hand to the rest position. When a trial is completed successfully, the monkey would receive water rewards. The training durations are 2~3 months before the monkeys successfully perform the tasks.

### 2.2. Neural Data Acquisition

The neural data are collected from the Utah microelectrode array (Blackrock, 96 channels) chronically implanted in the hand area of PMd, contra-lateral to the trained hand (left hemisphere for B03 and right hemisphere for B04). The surgical procedures are the same as described in [19]. All experimental procedures in this study conformed to the Guide for the Care and Use of Laboratory Animals (China Ministry of Health) and were approved by the Animal Care Committee at Zhejiang University, China.

Neural activities are recorded by the Cerberus data acquisition system (Blackrock, USA). Signals were amplified and analog-filtered by the Butterworth band-pass filter at 0.3–7500 Hz and further digitized (14 bit resolution, 30 kHz sample rate) and digitally filtered (Butterworth high pass filter) at 250 Hz. The spike activities were detected from the filtered signal by a threshold value method (the threshold was −5.5 times of the root mean square of the baseline signal). And the spike timings were recorded. Spike activities were sorted by Offline Sorter (Plexon, USA). Different spike waveforms were discriminated by a time-amplitude discriminator and a principle component analysis (PCA) algorithm [20]. Each neuron was identified by observing the spike waveforms and the channel locations based on the above spike sorting method. In addition, the event timings of Light ON, Light OFF, and rewarding were also recorded synchronously via the digital input port of the Cerberus system. 

### 2.3. Data Analysis

To analyze neural activities in the two tasks, we mainly focus on the period from the resting state to stretch out, grasp, and hold. For each trial, we extract 0.5 s before Light ON to 1.7 s after Light ON for Monkey B03 and 1.3 s after Light ON for B04 (B04 moved faster than B03). 

For the desired period, neurons' firing rates are binned in a 100 ms window. Firstly, a one-way ANOVA test is applied to observe whether the neurons fire significantly different from the rest state when performing the tasks. Then a quantitative method, mutual information, is introduced to measure the information amount between the neural firings and the target task. In order to directly exploit the timing of the neurons' firing, we examine the spike indicator (whether there is a spike or not) every 10 ms. The mutual information between the neural activities and the task (reaching or grasping) is defined as

(1) I(spk;y)=∑y=y1,y2,y3,y4p(y)∑spk=0,1p(spk ∣ y)∗log⁡2⁡(p(spk ∣ y)p(spk)),

where *y*_1_, *y*_2_, *y*_3_, and *y*_4_ represent four different actions in either task, and *p*(*y*) represents the probability of the corresponding action (in our experiments, it is 1/4 due to the same trial numbers); *p*(spk | *y*) represents the conditional probability of the corresponding firing rate (0 or 1) for a certain action; and *p*(spk) represents the probability of the corresponding firing rate of the target task. Please refer to [21, 22] for details. We calculate the cumulative sum of the neurons' MI according to a descending order for each task. When the cumulative sum of the MI reaches 90 percent of the total amount, the last MI that has been added is regarded as the threshold for the task. The threshold is applied to divide the neurons into the tuned ones and nontuned ones and calculated session by session, respectively.

An SVM is implemented as a decoder to quantify the contribution of the tuned neurons to the BMI decoding. Firstly, a subset of the tuned neurons according to the thresholds described above is used in classification, compared with the decoding by full ensemble, respectively, for the two tasks. Noticing that there might be overlaps between the neurons tuned to reaching and the neurons tuned to grasping, that is, the neurons tuned to reaching as well as grasping, we separate them from the neurons tuned to one property only and compare the decoding performance including or excluding them.

An SVM model maps the neural data into a high-dimension space by a kernel function, and different categories are divided by a hyper plane. For a specific SVM model, the hyper plane is optimized by (2), according to the statistical learning theory [23]:

(2) min⁡u,b,εi ⁡12||u||2+C∑t=1Nεtsubject  to: yt(uTXt+b)≥1−εt,t=1,…,N; εt≥0, t=1,…,N,

where *y*_*t*_ represents the category of different movements, and *X*_*t*_ represents the neural firing rate, which is an *n* by *m* vector (*n* is the number of neurons in a session, and *m* is the number of bins). In our experiment, *m*  is 18 for B03 and 14 for B04. The parameters *u* and *b* indicate the normal vector of a hyper plane and its offset. The goal of (2) is to find an optimal separation plane which is farthest away from the nearest neural data in both classes. The parameter *ε*_*t*_ is a dummy variable. The regularization term makes sure that the neural data in the training set is misclassified with a cost, because there is noise and other measurement errors. The parameter *C* is used for controlling the balance between the overtraining and the generalization in testing. In our experiment, we take radial basis function as the kernel function. And the parameters of the radial function *γ* and *C* are determined by a 2-fold cross validation. The algorithm is implemented in MATLAB using open source library LIBSVM [24]. We would like to remark at this point that SVM is not the only option to evaluate the decoding performance. Any effective classifier may work here.

## 3. Results

The goal of this work is to find out how a certain neuron in the PMd area is tuned to reaching and grasping, and this section shows some example neurons with different tuning characteristics, followed by the quantitative analysis of the neurons' tuning properties for different tasks; and the last step is to verify the contribution of the tuned neurons to the BMI decoding.

5 sessions of neural signals for each monkey were obtained during a period of half a month. In each session, monkeys were required to do both Reaching task (100 trials divided in 25 trials for each reaching direction) and Grasping task (200 trials divided in 50 trials for each grasping gesture). For Monkey B03, neural data were recorded from 96 channels, and for Monkey B04, neural data were recorded from 64 channels. After offline sorting, 22–47 neurons (from B03) and 30–66 neurons (from B04) were isolated in each session.

### 3.1. Observing Neurons' Tuning Activities in Time

**Figure 2:**
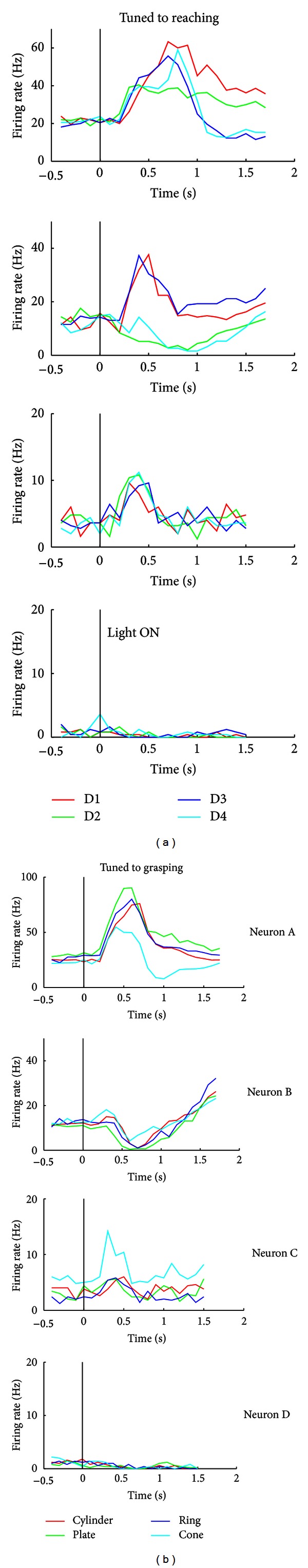
The tuning activities of four example neurons from both monkeys during the Reaching and the Grasping tasks. The *x-*axis represents the times (in seconds) from the rest state (about 0.4-0.5 s) to the movement state (about 1.4–1.7 s). The *y*-axis represents the firing rate (in Hz). The vertical line in each panel represents the time of Light ON, which is defined as 0 s. Each row shows the variation of the same neuron's firing rates in the two tasks (left: the Reaching task; right: the Grasping task). Neurons A and B are from the first session of B03, and Neurons C and D are from the third session of B04. The four different colors in one plot represent four different actions of a task. Specifically, in the Reaching task, the red color indicates reaching to the direction of upper-left corner (D1), the green color indicates reaching to the direction of upper-right corner (D2), the blue color indicates reaching to the direction of lower-left corner (D3), and the light blue color indicates reaching to the direction of lower-right corner (D4). In the Grasping task, the red color indicates grasping the cylinder, the green color indicates grasping the plate, the blue color indicates grasping the ring, and the light blue color indicates grasping the cone.

To give an intuitively view of the neurons' tuning characteristic in the Reaching and the Grasping tasks, we average the firing rates across trials in one session for each task. Here, we plot four example neurons (Figure 2) which show different firing patterns during the tasks. The average firing rates in each task are aligned by the time of Light ON and plotted in the same figure. Neurons A and B are selected from one session of B03, and Neurons C and D are selected from one session of B04. We can see that Neuron A fires significantly higher than the baseline in the two tasks. And the four curves both in the Reaching task (corresponding to the four reaching directions) and the Grasping task (corresponding to the four grasping gestures) are scattered. Therefore, we consider Neuron A as tuned to both tasks. For Neuron B, the firing curves in the Reaching task can be discriminated but grouped together in the Grasping task. We consider it as tuned to reaching only. The firing pattern of Neuron C is just contrary to Neuron B, and we regarded it as tuned to grasping only. And for Neuron D, which fires at the baseline level in both tasks, is regarded as no-tuning.

To inspect whether the neurons' tuning properties commonly exist or not, a one-way ANOVA test (*P* < 0.05) has been carried out [25]. Task-related responses of each neuron are statistically assessed by comparing the firing rates between the movement state and the rest state. In the reaching task, 80.96% of the neurons from B03 show significant difference in the firing rate relative to the baseline. The percentage is 72.41% for B04. These neurons are classified as reaching-related ones. In the Grasping task, the percentages are 88.02% and 86.59%, respectively, for B03 and B04. These neurons are classified as grasping-related ones (one-way ANOVA, *P* < 0.05). Among the reaching-related neurons, a large fraction (84.04% for B03 and 68.26% for B04) further shows reaching tuning property (the firing rates for at least one reaching direction in at least two bins were significantly different to the others). This situation is the same as the grasping-related neurons, 92.03% and 73.88% for B03 and B04 show grasping tuning property, respectively, (one-way ANOVA, *P* < 0.05). These results show that a majority of the neurons show tuning property to the Reaching and the Grasping tasks.

### 3.2. Mutual Information Analysis

To evaluate the neurons' tuning characteristic quantitatively, the mutual information (MI) between each neuron and the corresponding task is calculated. For each neuron there are two MI values respectively for the reaching and the grasping task.

**Figure 3:**
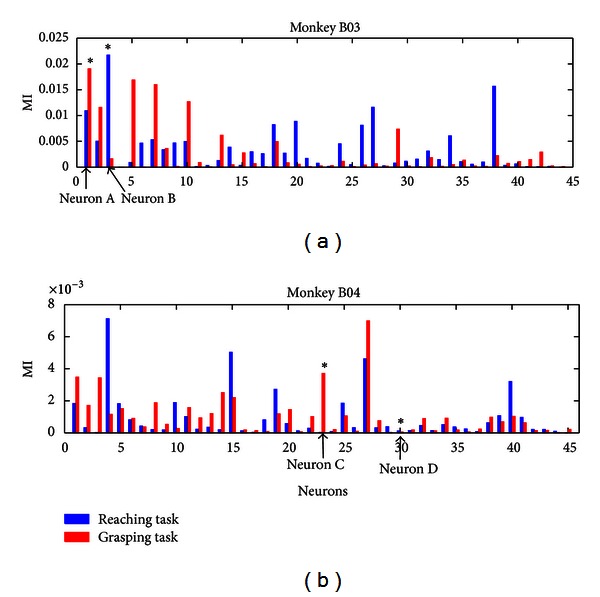
The mutual information in one session for the two tasks. The *x-*axis represents the neurons, and the *y-*axis represents the MI values in the Reaching and the Grasping tasks. The blue bars represent the Reaching task, and the red bars represent the grasping task. The upper panel is for Monkey B03, and the bottom one is for B04. The asterisks represent the four neurons shown in Figure 2, which are instructed by four arrows under the *x-*axis.

Figure 3 displays the MI values for the two tasks in one session. The MI values in the two tasks reflect some characteristics. Some neurons exhibit large MI in reaching (blue bar) while very small MI in grasping (red bar), suggesting that they are more sensitive to the reaching task. By contrast, some display large MI in grasping while small MI in reaching, suggesting they are involved in grasping. Besides, there are also some neurons presenting large MI in both tasks, which indicates that they are related to both conditions. 

Note that the four example neurons shown in Figure 2 are marked by the asterisks in the above figure, and the corresponding neuron signs are instructed by the arrows under the *x-*axis. Neuron A, ranking first in the upper panel, which is regarded as tuned to both conditions in Figure 2, shows large MI values in both tasks. Neuron B, which tuned to reaching only, exhibits large MI in reaching while small MI in grasping. Neuron C, ranking 23 in the bottom panel, displays large MI only in grasping. And Neuron D shows small MI in both conditions. This demonstrates that a neuron's tuning to a task reveals large MI in the corresponding task, and the MI can indicate a neuron's tuning property. 

A threshold method on MI is employed to quantitatively evaluate a neuron's tuning property. If the MI values in reaching and grasping exceed the thresholds of both tasks, the neuron is tuned to both conditions. If the MI of one task is greater than the task's threshold but the MI of another task is below the task's threshold, the neuron is defined as tuned to one condition only.  Table 1 shows the contribution of the tuned neurons in the corresponding task, that is, the percentage of the information provided by the tuned neurons, which are averaged across five sessions. The number of the corresponding tuned neurons is also given in the brackets. Take Monkey B03 as an example, in the Reaching task, the information provided by the neurons tuned only to reaching and those tuned to both conditions, respectively, accounts for 30.74% and 54.81%. And in the Grasping task, the proportion is 27.77% and 60.94%, respectively, for the neurons tuned only to grasping and the neurons tuned to both conditions. The information distribution for Monkey B04 is similar to B03.

It is interesting to notice that the contribution of the neurons tuned to both conditions is much larger than that of the neurons tuned to only one property in both tasks. One possible reason may be the number of the neurons tuned to both conditions is averagely greater than the number of the neurons tuned to one property only. The reveal of the large number of the neurons that tuned to both conditions is consistent with the study [12] that shows there exist PMd neurons that tuned to grasping as well as reaching.

### 3.3. Decoding Verification

**Figure 4:**
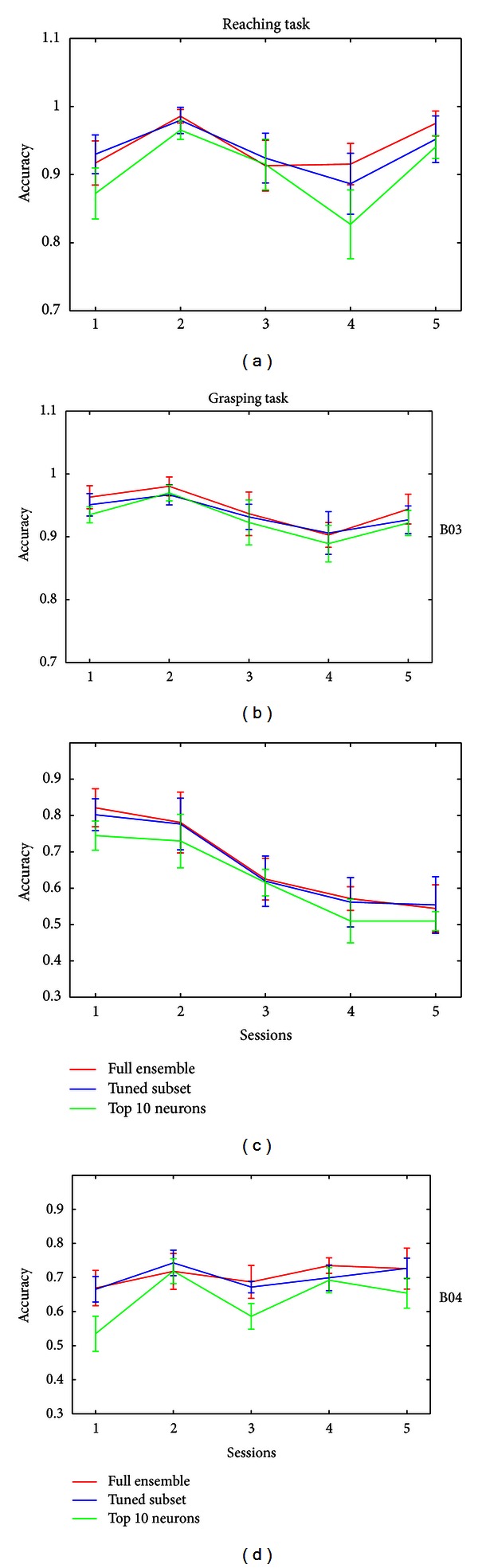
The decoding results by full ensemble versus the tuned neuron subset versus the top 10 well-tuned neurons. The *x-*axis represents the sessions, and the *y-*axis represents the decoding accuracy. The red line represents the decoding by full ensemble, the blue line represents the decoding by the tuned neuron subset, and the green line represents the decoding by the top 10 well-tuned neurons. The top two panels show the decoding in the Reaching (left) and the Grasping tasks (right) for Monkey B03. The bottom panels are for B04.

To verify the correlation between the neurons' tuning property and the tasks, we adopt the SVM to check the decoding performance by full ensemble versus the tuned neuron subset versus the top 10 well-tuned neurons. The decoding results are depicted in Figure 4. 

Compared with the decoding by full ensemble, the tuned neuron subset achieves quite close accuracy in both tasks for the two monkeys, and even using the top 10 well-tuned neurons can get a comparable performance. These results suggest that the tuned neurons contain the majority of the information related to the corresponding task, and even a small subset of well-tuned neurons is able to achieve an excellent decoding performance.

In Figure 4, the tuned neuron subset in each task contains two types of tuned neurons, that is, the neurons tuned to one property only and the neurons tuned to both conditions. To investigate the role of the neurons tuned to both conditions, we further compare the decoding by adding them into the neurons tuned only to one property. 

**Figure 5:**
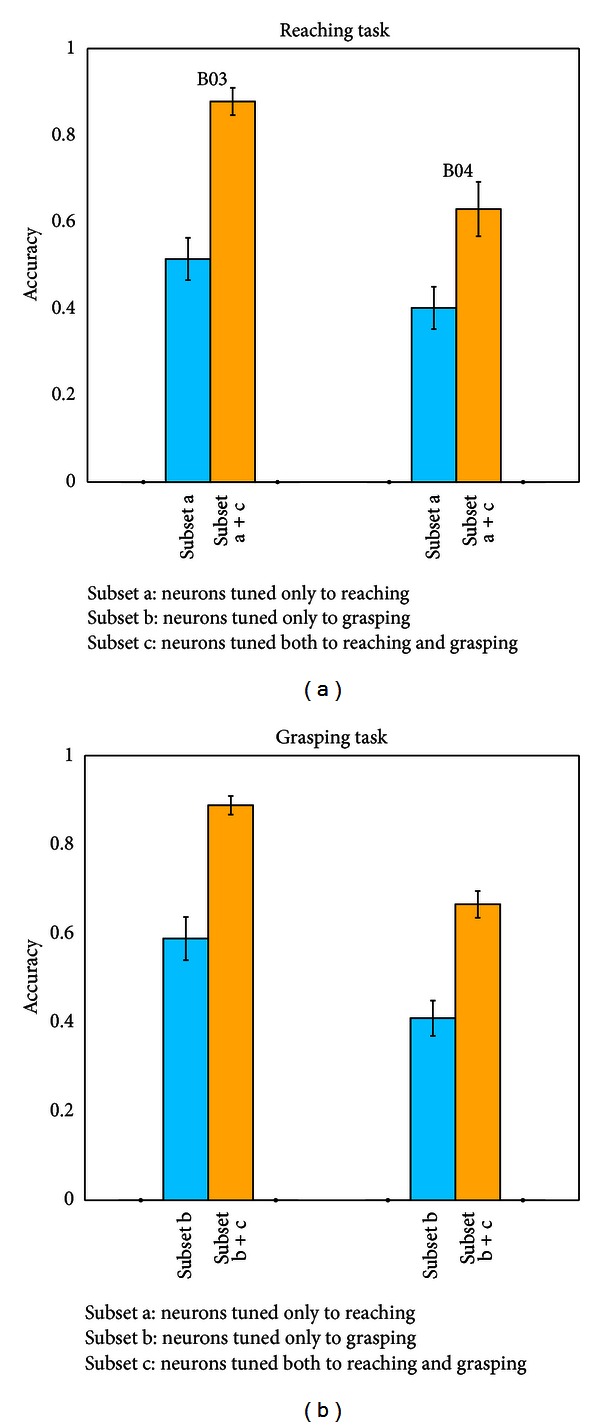
The decoding performance by different neuron subsets. The *y-*axis represents the decoding accuracy. The blue bars represent the decoding by the subset tuned to one property only, and the yellow bars represent the decoding by two types of tuned neurons (the subset tuned to one property only plus the subset tuned to both conditions). The left panel is for the Reaching task and the right panel for the Grasping task. In each panel, the left group is for B03 and the right group for B04. The subset a represents the neurons tuned only to reaching, the subset b represents the neurons tuned only to grasping, and the subset c represents the neurons tuned to both conditions.

Figure 5 includes three neurons subsets, namely, the subset a, the subset b, and the subset c, representing, respectively, the neurons tuned only to reaching, the neurons tuned only to grasping, and the neurons tuned to both conditions. The light blue bars represent the decoding by the subset tuned only to one property (i.e., the subset a in the Reaching task and the subset b in the Grasping task), and the yellow bars represent the decoding by two types of tuned neurons (the neurons tuned to one property only plus the neurons tuned to both conditions, that is, the subset a plus the subset c in the Reaching task and the subset b plus the subset c in the Grasping task). Adding the subset c to the subset a or the subset b, the decoding accuracy significantly increases both in the Reaching and the Grasping tasks, and the improvement is about 50.9%–70.6%. For the significant decoding performance improvement, one possible reason is the greater number of the neurons for the combination of the two types of tuned neurons. Another possibility is that the tuned neurons in the combined group make a better integration of the neuron circuits that can form a complete and more complicated reaching and grasping action. Hoshi and Tanji in their reviews have demonstrated that the neurons in PMd receive multiple aspects of motor information (including arm use, target location, and instructed movement direction) that encodes in a circuit to form an appropriate action [26–29]. These studies indicate that the PMd has played a major role in integrating multi-information to formulate a complicated movement. Therefore, the combined groups of the tuned neurons may make a better integration of different information that eventually achieves a precise prediction of the output movement.

## 4. Conclusion and Discussion

In the current work, we have studied the tuning characteristic of the neurons in the PMd area in reaching and grasping, respectively. We design two BMI behavior paradigms which keep one factor constant but change another. The first condition is a “Reaching task” which requires the monkeys to reach in four different directions but grasp the same object. The other condition is a “Grasping task” which requires the monkeys to grasp four different objects but reach in the same direction. We propose to utilize mutual information (MI) to quantitatively evaluate the neurons' tuning property in both tasks. We find that there exist neurons in PMd that are tuned only to reaching, tuned only to grasping, and tuned to both tasks. When applied with a support vector machine (SVM), the movement decoding accuracy by the tuned neuron subset in either task is quite close to the performance by full ensemble. Our results demonstrate the diversity of neural tuning to reaching and grasping in the PMd area. The tuning characteristic of the PMd neurons in the Reaching and the Grasping tasks can be significant. The tuned neurons contain more information related to the movement.

An interesting phenomenon is that the MI of the neurons tuned to both conditions is larger than that of the neurons tuned to one property only in both tasks. One possible reason may be that the number of the neurons tuned to both conditions is greater than the number of the neurons tuned to one property only. The reveal of the large number of the neurons that tuned to both conditions is consistent with the study [12] that shows there exist PMd neurons that tuned to grasping as well as reaching. The larger number may also cause improvement of the decoding performance when combining such neurons tuned to both conditions with the subset tuned to one property only. Furthermore, the tuned neurons in the combined group may make a better integration of the neuron circuits that can form a more complicated reaching and grasping action, which can contribute to the better decoding of multimotor information from PMd neurons. The mechanism of neural activities for the reaching or grasping task requires further study.

## Figures and Tables

**Table 1:** The percentage of the information provided by the tuned neurons in the Reaching and the Grasping task. The number shown in the brackets represents the corresponding tuned neurons.

Monkey	The contribution of the tuned neurons in the two tasks			
	Reaching task		Grasping task	
	Tuned only to reaching (averaged number of neurons)	Tuned to both conditions (averaged number of neurons)	Tuned only to grasping (averaged number of neurons)	Tuned to both conditions (averaged number of neurons)
B03	30.74% (7)	54.81% (9)	27.77% (5)	60.94% (9)
B04	23.16% (9)	65.51% (14)	30.4% (9)	57.89% (14)

## References

[1] Hochberg LR, Serruya MD, Friehs GM (2006). Neuronal ensemble control of prosthetic devices by a human with tetraplegia. *Nature*.

[2] Carmena JM, Lebedev MA, Crist RE (2003). Learning to control a brain-machine interface for reaching and grasping by primates. *PLoS Biology*.

[3] Lebedev MA, Carmena JM, O’Doherty JE (2005). Cortical ensemble adaptation to represent velocity of an artificial actuator controlled by a brain-machine interface. *Journal of Neuroscience*.

[4] Velliste M, Perel S, Spalding MC, Whitford AS, Schwartz AB (2008). Cortical control of a prosthetic arm for self-feeding. *Nature*.

[5] Hochberg LR, Bacher D, Jarosiewicz B (2012). Reach and grasp by people with tetraplegia using a neurally controlled robotic arm. *Nature*.

[6] Wessberg J, Stambaugh CR, Kralik JD (2000). Real-time prediction of hand trajectory by ensembles of cortical neurons in primates. *Nature*.

[7] Taylor DM, Tillery SIH, Schwartz AB (2002). Direct cortical control of 3D neuroprosthetic devices. *Science*.

[8] Artemiadis PK, Shakhnarovich G, Vargas-Irwin C, Donoghue JP, Black MJ Decoding grasp aperture from motor-cortical population activity.

[9] Vargas-Irwin CE, Shakhnarovich G, Yadollahpour P, Mislow JMK, Black MJ, Donoghue JP (2010). Decoding complete reach and grasp actions from local primary motor cortex populations. *Journal of Neuroscience*.

[10] Hao Y, Zhang Q, Zhang S (2013). Decoding grasp movement from monkey premotor cortex for real-time prosthetic hand control. *Chinese Science Bulletin*.

[11] Saleh M, Takahashi K, Hatsopoulos NG (2012). Encoding of coordinated reach and grasp trajectories in primary motor cortex. *Journal of Neuroscience*.

[12] Stark E, Asher I, Abeles M (2007). Encoding of reach and grasp by single neurons in premotor cortex is independent of recording site. *Journal of Neurophysiology*.

[13] Townsend BR, Subasi E, Scherberger H (2011). Grasp movement decoding from premotor and parietal cortex. *Journal of Neuroscience*.

[14] Burnod Y, Grandguillaume P, Otto I, Ferraina S, Johnson PB, Caminiti R (1992). Visuomotor transformations underlying arm movements toward visual targets: a neural network model of cerebral cortical operations. *Journal of Neuroscience*.

[15] Messier J, Kalaska JF (2000). Covariation of primate dorsal premotor cell activity with direction and amplitude during a memorized-delay reaching task. *Journal of Neurophysiology*.

[16] Raos V, Franchi G, Gallese V, Fogassi L (2003). Somatotopic organization of the lateral part of area F2 (dorsal premotor cortex) of the macaque monkey. *Journal of Neurophysiology*.

[17] Raos V, Umiltá MA, Gallese V, Fogassi L (2004). Functional properties of grasping-related neurons in the dorsal premotor area F2 of the macaque monkey. *Journal of Neurophysiology*.

[18] Dum RP, Strick PL (2005). Frontal lobe inputs to the digit representations of the motor areas on the lateral surface of the hemisphere. *Journal of Neuroscience*.

[19] Zhang QS, Zhang SM, Hao YY (2012). Development of an invasive brain-machine interface with a monkey model. *Chinese Science Bulletin*.

[20] Nicolelis MAL, Ghazanfar AA, Faggin BM, Votaw S, Oliveira LMO (1997). Reconstructing the engram: simultaneous, multisite, many single neuron recordings. *Neuron*.

[21] Wang Y, Principe JC, Sanchez JC (2009). Ascertaining neuron importance by information theoretical analysis in motor Brain-Machine Interfaces. *Neural Networks*.

[22] Wang Y, Principe JC (2010). Instantaneous estimation of motor cortical neural encoding for online brain-machine interfaces. *Journal of Neural Engineering*.

[23] Vapnik VN (1998). *Statistical Learning Theory*.

[24] Chang CC, Lin CJ (2011). LIBSVM: a Library for support vector machines. *ACM Transactions on Intelligent Systems and Technology*.

[25] Umilta MA, Brochier T, Spinks RL, Lemon RN (2007). Simultaneous recording of macaque premotor and primary motor cortex neuronal populations reveals different functional contributions to visuomotor grasp. *Journal of Neurophysiology*.

[26] Hoshi E, Tanji J (2007). Distinctions between dorsal and ventral premotor areas: anatomical connectivity and functional properties. *Current Opinion in Neurobiology*.

[27] Hoshi E, Tanji J (2004). Functional specialization in dorsal and ventral premotor areas. *Progress in Brain Research*.

[28] Mitz AR, Godschalk M, Wise SP (1991). Learning-dependent neuronal activity in the premotor cortex: activity during the acquisition of conditional motor associations. *Journal of Neuroscience*.

[29] Grafton ST, Fagg AH, Arbib MA (1998). Dorsal premotor cortex and conditional movement selection: a PET functional mapping study. *Journal of Neurophysiology*.

